# KNOW-CKD (KoreaN cohort study for Outcome in patients With Chronic Kidney Disease): design and methods

**DOI:** 10.1186/1471-2369-15-80

**Published:** 2014-05-19

**Authors:** Kook-Hwan Oh, Sue Kyung Park, Hayne Cho Park, Ho Jun Chin, Dong Wan Chae, Kyu Hun Choi, Seung Hyeok Han, Tae Hyun Yoo, Kyubeck Lee, Yong-Soo Kim, Wookyung Chung, Young-Hwan Hwang, Soo Wan Kim, Yeong Hoon Kim, Sun Woo Kang, Byung-Joo Park, Joongyub Lee, Curie Ahn

**Affiliations:** 1 Department of Internal Medicine, Seoul National University, 101 Daehakro, Chongno Gu, Seoul 110-744, Korea; 2 Department of Preventive Medicine, Seoul National University College of Medicine, Seoul, Korea; 3 Department of Internal Medicine, Yonsei University, Severance Hospital, Seoul, Korea; 4 Department of Internal Medicine, Kangbuk Samsung Medical Center, Sungkyunkwan University, Seoul, Korea; 5 Department of Internal Medicine, The Catholic University of Korea, Seoul St. Mary’s Hospital, Seoul, Korea; 6 Department of Internal Medicine, Gachon University, Gil Hospital, Incheon, Korea; 7 Department of Internal Medicine, Eulji University, Eulji General Hospital, Seoul, Korea; 8 Department of Internal Medicine, Chonnam National University Medical School, Gwangju, Korea; 9 Department of Internal Medicine, Inje University, Pusan Paik Hospital, Busan, Korea; 10 Medical Research Collaborating Center, Seoul National University Hospital and Seoul National University College of Medicine, Seoul, Korea

**Keywords:** KNOW-CKD, Chronic kidney disease, Cohort, Etiology, Progression, Complication, Natural course

## Abstract

**Background:**

The progression and complications of chronic kidney disease should differ depending on the cause (C), glomerular filtration rate category (G), and albuminuria (A). The KNOW-CKD (KoreaN Cohort Study for Outcome in Patients With Chronic Kidney Disease), which is a prospective cohort study, enrolls subjects with chronic kidney disease stages 1 to 5 (predialysis).

**Methods/Design:**

Nine nephrology centers in major university hospitals throughout Korea will enroll approximately 2,450 adults with chronic kidney disease over a 5-year period from 2011 to 2015. The participating individuals will be monitored for approximately 10 years until death or until end-stage renal disease occurs. The subjects will be classified into subgroups based on the following specific causes of chronic kidney disease: glomerulonephritis, diabetic nephropathy, hypertensive nephropathy, polycystic kidney disease, and others. The eligible subjects will be evaluated at baseline for socio-demographic information, detailed personal/family history, office BP, quality of life, and health behaviors. After enrollment in the study, thorough assessments, including laboratory tests, cardiac evaluation and radiologic imaging, will be performed according to the standardized protocol. The biospecimen samples will be collected regularly. A renal event is defined by >50% decrease in estimated GFR (eGFR) from the baseline values, doubling of serum creatinine, or end-stage renal disease. The primary composite outcome consists of renal events, cardiovascular events, and death. As of September 2013, 1,470 adult chronic kidney disease subjects were enrolled in the study, including 543 subjects with glomerulonephritis, 317 with diabetic nephropathy, 294 with hypertensive nephropathy and 249 with polycystic kidney disease.

**Discussion:**

As the first large-scale chronic kidney disease cohort study to be established and maintained longitudinally for up to 10 years, the KNOW-CKD will help to clarify the natural course, complication profiles, and risk factors of Asian populations with chronic kidney disease.

**Trial registration:**

No. NCT01630486 at http://www.clinicaltrials.gov.

## Background

Chronic kidney disease (CKD) is a rapidly growing disease worldwide. The prevalence of CKD in Korea is estimated to be 13% in the adult population [1]. Approximately 5% of the Korean adult population is estimated to have decreased renal function with an estimated glomerular filtration rate (eGFR) less than 60 ml/min per 1.73 m^2^[1]. CKD, which leads to dialysis or kidney transplantation, presents a major threat to cardiovascular (CV) events and all-cause mortality [2,3]. A patient with CKD is more likely to die of CV disease than to start renal replacement therapy.

Since 2002, a large number of epidemiologic studies, in addition to the general population, have demonstrated a significantly increased risk of adverse consequences in subjects with albuminuria, even in the presence of normal or mildly decreased GFR [4,5]. This finding warrants a broader view of the early stages of CKD within the nephrology population to determine the variables associated with CKD and the increased risks of death and adverse outcomes [6]. However, the mechanistic link between albuminuria and adverse consequences has not been established.

Kidney Disease: Improving Global Outcomes (KDIGO) 2012 Clinical Practice Guideline for the Evaluation and Management of CKD recommends a new classification of CKD, based on the cause, GFR category, and albuminuria (CGA) [7]. The characteristics of CKD differ significantly depending on the underlying disease or the location of the pathologic-anatomic abnormalities. Therefore, we hypothesized that the progression and complications of CKD should be different among the disease subgroups, which cause CKD. A new perspective in the viewpoint of the “CGA” paradigm is required to analyze and interpret the various parameters associated with CKD.

Nevertheless, recently established CKD cohort studies [8-11] are primarily based on the CKD population with low GFR (less than 60 ml/min per 1.73 m^2^). The above studies targeted the cardiovascular complications from subjects with moderate or severe renal impairment. In these studies, the early stages of renal injury are excluded from their target population and the specific causes of CKD are not considered. In contrast, a new CKD cohort study, including all of the stages of the pre-dialysis CKD population and considering the specific causes of CKD, may lead us to gain insight into understanding the natural progression of CKD and the association between CKD and CV outcomes or mortality.

The KNOW-CKD (KoreaN Cohort Study for Outcome in Patients With Chronic Kidney Disease), funded by the Korea Centers for Disease Control and Prevention (KCDC), was established in 2011 by a group of nephrologists, epidemiologists, biostatisticians, and clinical pathologists in Korea.

The goals of the KNOW-CKD are as follows: 1) to establish an adult pre-dialysis CKD cohort covering the early stages of CKD, as well as the advanced stages of CKD, 2) to investigate the natural history of CKD, such as renal progression, death, and complications with respect to the CGA categories, 3) to evaluate the etiologic factors for renal progression and related complications, 4) to identify the mechanistic link between renal injury and adverse outcomes, and 5) to identify the genetic and molecular influences on renal progression and complications.

In the present paper, we report our study design and methods and results from study participants registered from 2011 to 2013.

## Methods/Design

### Organization

The KNOW-CKD is a patient-based cohort study. Nephrologists working in nine clinical centers in the major university-affiliated hospitals and epidemiologists, pathologists and biostatisticians in a research modulating center are participating in this study. The basic protocol of the study was approved by the ethical committee of each participating center including Institutional Review Boards of Seoul National University Hospital, Severance Hospital, Kangbuk Samsung Medical Center, Seoul St. Mary’s Hospital, Gil Hospital, Eulji General Hospital, Chonnam National University Hospital and Pusan Paik Hospital. Among the nine clinical centers, six centers are located in the metropolitan city of Seoul, one in a satellite area of Seoul, Gyeonggi-do (a province), and two in the southern area of Korea (i.e., Chonnam-do and Busan). Throughout the national health care system, Korea has 44 tertiary-care general hospitals. The distribution of our participating clinical centers is parallel to the distribution of the major hospitals in Korea. The study is supervised by the CKD Advisory Committee, which comprises members from the KCDC and the Korean Society of Nephrology (KSN) (NCT01630486 at http://www.clinicaltrials.gov).

### Study population

Each participating center will enroll approximately 250 consecutive individuals over a 5-year period from 2011 until 2015, totaling 2,450 adult patients with CKD who provide written informed consent. The participating individuals will be monitored for approximately 10 years until death or until ESRD occurs.

The KNOW-CKD will enroll ethnic Korean patients with CKD who range in age between 20 years and 75 years. The CKD stages from 1 to 5 (predialysis), based on the eGFR, is calculated using the four-variable Modification of Diet in Renal Disease (MDRD) equation as follows:

eGFR (ml/min per 1.73 m^2^) = 175 × [serum Cr (mg/dl)] ^-1.154^ × [age]^-0.203^ × [0.742 if female] × [1.212 if black], using serum creatinine concentrations measured at a central laboratory and an assay traceable to the international reference material [12].

Excluded subjects are those who 1) are unable or unwilling to give written consent, 2) have previously received chronic dialysis or organ transplantation, 3) have heart failure (NYHA class 3 or 4) or liver cirrhosis (Child-Pugh class 2 or 3), 4) have a past or current history of malignancy, 5) are currently pregnant, or 6) have a single kidney due to trauma or kidney donation.

We defined and allocated the specific causes of the CKD into four subgroups: glomerulonephritis (GN), diabetic nephropathy (DN), hypertensive nephropathy (HTN), and polycystic kidney disease (PKD). The definition of the subgroup is defined by the pathologic diagnosis, in the event that the biopsy result is available. Otherwise, the subgroup classification depends on the clinical diagnosis. GN is defined by the presence of glomerular hematuria or albuminuria with or without an underlying systemic disease causing glomerulonephritis. The diagnosis of DN is based on albuminuria in a subject with type 2 diabetes mellitus and the presence of diabetic retinopathy. HTN is defined by the patient’s hypertension history and the absence of a systemic illness associated with renal damage. Unified ultrasound criteria [13] will be used to diagnose PKD. The other causative diseases will be categorized as ‘unclassified’.

### Data collection

The detailed protocol for data and sample collection is described in Table 1. The eligible subjects will be screened and evaluated at baseline for socio-demographic information, smoking, alcohol consumption, medication, and detailed personal and family medical histories. The anthropometric measurements (height, weight, and waist/hip ratios), and measurements of the resting blood pressure (BP) in the clinic and pulse pressure using an electronic sphygmomanometer will be conducted. The subjects will complete questionnaires concerning quality of life, socio-economic status, educational level, physical activity, health behaviors, and health care facility utilization. After enrollment in the study, the laboratory tests, cardiac evaluation and radiologic imaging will be performed according to the specific protocol. DNA, serum and urine samples will be initially collected. Serum and urine samples will be subsequently collected on a regular basis according to the standardized protocol. The details regarding the study visit and test schedule are shown in Table 1.

**Table 1:** Schedule of the KNOW-CKD at each visit

**Parameter**	**Visit time (year)**												
	**Screening**	**Baseline**	**0.5**	**1**	**2**	**3**	**4**	**5**	**6**	**7**	**8**	**9**	**10**
Informed consent	O												
Demographic information	O												
Medical History	O												
Eligibility confirmation	O												
Recent events			O	O	O	O	O	O	O	O	O	O	O
Medications		O	O	O	O	O	O	O	O	O	O	O	O
Health Questionnaires		O						O					
KDQOL questionnaires		O						O					
Blood pressure		O	O	O	O	O	O	O	O	O	O	O	O
Anthropometry		O	O	O	O	O	O	O	O	O	O	O	O
CBC, chemistry		O	O	O	O	O	O	O	O	O	O	O	O
IDMS-traceable Cr, MDRD eGFR		O	O	O	O	O	O	O	O	O	O	O	O
Cystatin C		O	O	O	O	O	O	O	O	O	O	O	O
HbA1c (diabetic subjects)		O	O	O	O	O	O	O	O	O	O	O	O
Lipid panel, CRP, iron profile		O		O		O		O		O		O	
Intact PTH, 25D, 1,25 D, cardiac troponin T		O		O		O		O		O		O	
Urinalysis		O	O	O	O	O	O	O	O	O	O	O	O
24-h urine collection		O				O		O		O		O	
Electrolyte/Cr/osmolality, albumin/protein (Spot urine)		O		O		O		O		O		O	
Electrocardiogram, chest X ray		O			O		O		O		O		O
Echocardiography, Pulse wave velocity, Ankle-brachial index, MDCT, LS spine lateral X-ray for vascular calcification		O					O				O		
Bone mineral density		O					O				O		O
DNA sample		O											
Plasma/urine sample		O	O	O	O	O	O	O	O	O	O	O	O
Fundus exam (diabetic subjects)		O											
Abdomen CT or MRI (polycystic kidney disease)		O			O		O		O		O		O
Family screen (polycystic kidney disease)		O											

CBC, complete blood count; KDQOL, Kidney Disease –Quality of Life; IDMS-serum cr, serum creatinine measurement using IDMS-traceable method; MDCT, multidetector computed tomography for coronary calcium score.

All of the data, including clinical information, laboratory results and outcome, will be entered into a web-based, electronic, case-reporting form (eCRF). The electronic data management system for the KNOW-CKD was developed by the division of data management in Seoul National University Medical Research Collaborating Center.

### Outcome variables

A renal event is defined by a >50% decrease in eGFR from the baseline values, doubling of serum creatinine, or ESRD. The estimated GFR for defining renal outcome will be based on the four-variable MDRD formula. However, the Chronic Kidney Disease Epidemiology Collaboration (CKD-EPI) creatinine equation [14] or the CKD-EPI creatinine-cystatin C equation [15] will also be employed to estimate GFR.

At each visit, the subjects will be queried and evaluated for CV events, including myocardial infarction (fatal and nonfatal), coronary revascularization, stroke and new onset or aggravation of congestive heart failure. The causes of death will be verified and classified as cardiac, non-cardiac and unknown. An independent committee will adjudicate all of the primary outcomes. The subjects who withdraw from the study will be traced for this type of information with the help of the National Health Insurance System and Korea Statistical Information Service. The secondary outcomes are hospitalization, fracture, QOL, and economic status during the follow-up.

### Biospecimen collection

Using an EDTA tube, 3 ml of whole blood will be obtained and sent immediately for extracting DNA to the central laboratory. Another 10 ml of whole blood will be obtained using the serum separation tube (SST) and centrifuged within 1 hour for serum separation and sent to the central laboratory for measurements of creatinine, cystatin C, intact parathyroid hormone (iPTH), 25-hydroxyvitamine D3 (25D), 1,25-dihydroxyvitamin D3 (1,25D), and troponin T. Serum creatinine will be measured using an IDMS-traceable method; and cystatin C will be calculated using immunonephelometry calibrated against the reference material [15-17]. The first-voided urine samples (15 ml) will be collected for assaying albumin, protein, calcium (Ca), phosphorus (P), urea, uric acid, electrolytes and osmolality at the central laboratory. The aliquots of the remaining serum and urine samples will be stored in a deep freezer (−70°C) in two different places for future studies. Stringent rules for the sample harvest and processing will be applied to ensure accurate assays. Blood and urine sampling will be conducted under strict monitoring guidelines throughout the study period.

### Follow-up

The participating subjects will visit the center according to the follow-up schedule. At each visit, the subjects will undergo scheduled tests; the investigators will review the subjects’ recent medical histories and occurrence of events. We assumed that 10% of the individuals would withdraw from the study and that ESRD or death would develop subsequently in 10% of the subjects annually [18]. Efforts will be made to prevent drop-outs, i.e., providing free medical examinations, frequent telephone calls and dietary education.

### **Institutional review board** (**IRB) approval**

The study protocol was approved by the IRB at each participating clinical center in 2011.

### Statistical power estimation

With an overall sample size of 600 subjects, which is the size of the each subgroup (GN, DN, HTN, and PKD), 80% power is achieved to detect hazard ratios of approximately 2.00 and 1.60 for an exposure with prevalence of 0.1 and 0.5 and a dropout rate of 10%.

### Statistical methods and plan

The baseline characteristics of the study participants for each subgroup were presented with descriptive statistics using a one-way analysis of variance or Kruskal-Wallis test according to the distribution of continuous variables and a chi-square test or Fisher’s exact test for categorical variables.

To analyze the association of outcome variables, the time-to-event analysis will be used to evaluate primary outcomes, such as renal events (initiation of renal replacement therapy and 50% decline of GFR), CV events (myocardial infarction and ischemic stroke) and death. The Kaplan-Meier curves and the proportional hazard models will serve as major statistical methods for the outcomes. The incidence or mortality rates according to each outcome variable will be calculated for each etiologic group; the incidence ratios will be presented with reference to the diabetic nephropathy subgroup. The effect of exposure on the development of outcomes will be analyzed separately in each group and in the overall groups. The heterogeneity of each relative risk within the KNOW-CKD subcohort will be analyzed using the Cochran Q test or using a shared-frailty model, where time-dependent confounders should be adjusted [19]. The CKD progression will be evaluated using decreased GFR calculations. Because it is common for a patient with CKD to have a non-linear decline in GFR or a prolonged period of non-progression [20], we will assess the longitudinal trajectory of GFR for the individual participants of KNOW-CKD using the Bayesian smoothing technique [21]. If the progression of CKD is steady and linear, the generalized estimating equation will be adopted to compare the slope of the GFR with repeated measurements. The progression of CKD will be supplemented with an event-based analysis, whereby the occurrence of ESRD or the substantial decrease of GFR will be treated as the events of interest.

### Results on interim analysis

**Figure 1:**
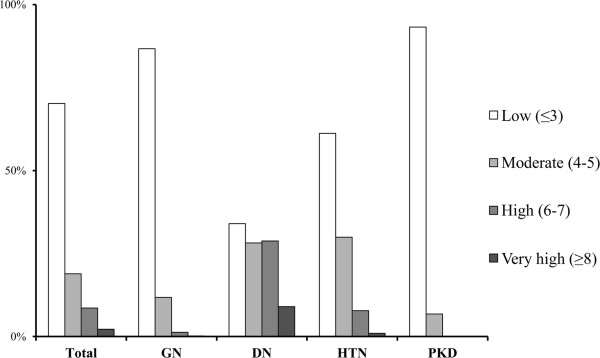
**Age-adjusted modified Charlson comorbidity index in total and subgroup participants, KNOW-CKD 2011–2013.** Abbreviations: GN, glomerulonephritis; DN, diabetic nephropathy; HTN, hypertensive nephropathy; PKD, polycystic kidney disease.

From July 2011 to September 2013, we enrolled 1,470 participants, including 543 GN, 317 DN, 294 HTN, 249 PKD sub-participants and remaining participants with unclassified subtypes. The baseline demographics and the laboratory values of KNOW-CKD are summarized in Tables 2 and 3. The mean age of the total CKD cohort at the time of study enrollment is 53.6 ± 12.3 years, 38.7% are female, and 31.6% diabetic. The proportion of each CKD stage is as follows; 10.9% (stage 1), 17.2% (stage 2), 18.2% (stage 3a), 22.3% (stage 3b), 23.7% (stage 4) and 7.6% (stage 5, nondialyzed). GN, DN, HTN and PKD were the most common causative diseases in descending order. At baseline, 7.3% of the cohort had a history of coronary heart disease, 8.5% had cerebrovascular disease, and 90.6% had hypertension. In terms of the Charlson comorbidity score and various cardiac parameters, the DN subgroup exhibited the worst profiles (Figure 1 and Table 2). In subjects with advanced CKD stages, the elevated levels of pulse pressure, serum phosphorus, and intact PTH were more pronounced than the subjects with early stages. Hemoglobin levels, 25D and 1,25D, were significantly lower and the left ventricular mass index (LVMI), coronary calcium score and valve calcification were higher in subjects with advanced CKD stages.

**Table 2:** General characteristics of study subjects participating in KNOW-CKD from 2011 to September, 2013

	**Total cohort**^**1**^	**Subcohort**				
		**GN**	**DN**	**HTN**	**PKD**	**p-value**^**2**^
Total participants from 2011 to 2013	**1470**	**543**	**317**	**294**	**249**	
Age	53.6 ± 12.3	50 ± 12.3	59.2 ± 9.3	59.6 ± 10.9	46.9 ± 10.4	< 0.001
Female	569 (38.7)	228 (42)	104 (32.8)	82 (27.9)	127 (51)	< 0.001
BMI (kg/m^2^)	24.4 ± 3.4	24.2 ± 3.4	25.2 ± 3.2	24.8 ± 3.5	23.5 ± 3.1	< 0.001
Waist-Hip ratio	0.89 ± 0.07	0.88 ± 0.07	0.92 ± 0.05	0.9 ± 0.07	0.88 ± 0.06	< 0.001
SBP (mmHg)	127.7 ± 16.5	123.2 ± 14.9	135.8 ± 18.7	127 ± 16.4	128 ± 13.5	< 0.001
DBP (mmHg)	76.6 ± 11.1	75.2 ± 10.6	76.1 ± 11.8	76.8 ± 11.6	79.8 ± 10.2	< 0.001
Pulse Pressure (mmHg)	51.1 ± 12.1	48 ± 10.6	59.7 ± 12.9	50.2 ± 11.5	48.2 ± 9.5	< 0.001
CKD stage 1	160 (10.9)	81 (14.9)	4 (1.3)	2 (0.7)	63 (25.3)	< 0.001
2	253 (17.2)	117 (21.5)	18 (5.7)	18 (6.1)	79 (31.7)	
3a	268 (18.2)	111 (20.4)	45 (14.2)	67 (22.8)	35 (14.1)	
3b	328 (22.3)	110 (20.3)	83 (26.2)	91 (31)	30 (12)	
4	349 (23.7)	93 (17.1)	121 (38.2)	95 (32.3)	30 (12)	
5	112 (7.6)	31 (5.7)	46 (14.5)	21 (7.1)	12 (4.8)	
Creatinine (mg/dL)	1.88 ± 1.17	1.67 ± 1.08	2.48 ± 1.38	2.12 ± 1.00	1.40 ± 0.98	< 0.001
Cystatin C (mg/L)	1.81 ± 0.95	1.61 ± 0.89	2.37 ± 0.99	2.04 ± 0.83	1.34 ± 0.79	< 0.001
eGFR (mL/min/1.73 m^2^)						
MDRD	48.6 ± 29.4	54.9 ± 30.6	32.7 ± 17.9	36.6 ± 17.4	67 ± 33.9	< 0.001
CKD-EPI cr (2009)	51.4 ± 30.3	58.3 ± 31	34 ± 19.5	37.9 ± 17.7	71.8 ± 33.6	< 0.001
Comorbid disease						
Coronary artery disease	107 (7.3)	22 (4.1)	44 (14.1)	32 (10.9)	3 (1.2)	< 0.001
Peripheral vascular disease	22 (1.5)	2 (0.4)	10 (3.2)	9 (3.1)	0 (0)	< 0.001
Cerebrovascluar disease	125 (8.5)	21 (3.9)	37 (11.9)	34 (11.6)	29 (11.6)	< 0.001
Diabetes	462 (31.6)	56 (10.4)	317 (100)	61 (20.7)	3 (1.2)	< 0.001
Hypertension	1325 (90.6)	470 (86.9)	302 (96.8)	283 (96.3)	213 (85.5)	< 0.001
Chronic heart failure	15 (1)	3 (0.6)	8 (2.6)	4 (1.4)	0 (0)	0.011
Arrhythmia	31 (2.1)	8 (1.5)	6 (1.9)	12 (4.1)	3 (1.2)	0.049
Cardiac parameters						
AAC score	1.2 ± 2.6	0.7 ± 1.9	2.6 ± 3.7	1.3 ± 2.7	0.3 ± 1.3	< 0.001
Mean baPWV (cm/s)	1525.0 ± 342.5	1396.4 ± 245.8	1819.2 ± 418.2	1569.1 ± 297.7	1392 ± 213.2	< 0.001
LVMI (g/m^2^) (Echocardiography)	95.0 ± 28.4	89.5 ± 25.2	106.5 ± 30.7	100.6 ± 31.9	85.9 ± 20.1	< 0.001
Coronary Ca score	191.7 ± 569.6	91.6 ± 409.9	496.3 ± 864.6	195.6 ± 569.5	28.5 ± 133.1	< 0.001
Troponin T (ng/mL)	0.016 ± 0.018	0.011 ± 0.010	0.030 ± 0.030	0.016 ± 0.011	0.008 ± 0.004	< 0.001
Valve calcification (Echocardiography)	262 (18.5)	105 (20.2)	82 (27.1)	65 (23)	7 (2.8)	< 0.001

^1^Total participants included those in 4 subgroups and the other unclassified kidney diseases.

ANOVA or Kruskal-Wallis test for continuous variables and Chi-square or Fisher’s Exact test for categorical variables.

Numbers denote mean ± s.d. or number (%).

*Abbreviations:* GN, glomerulonephropathy; DN , diabetic nephropathy; HTN, hypertensive nephropathy; PKD, autosomal dominant polycystic kidney disease; AAC, abdominal aorta calcification; baPWV, brachial-to-ankle pulse wave velocity; LVMI, left ventricular mass index.

**Table 3:** General characteristics of study participants according to CKD stage, KNOW-CKD from 2011 to September, 2013

**CKD Stage**^**1**^							
	**Stage 1**	**Stage 2**	**Stage 3a**	**Stage 3b**	**Stage 4**	**Stage 5**	**p-value**^**2**^
Total participants from 2011 to 2013	**160**	**253**	**269**	**328**	**350**	**112**	
Age	42.2 ± 11.8	49.6 ± 11.9	54.8 ± 11.7	56.7 ± 10.7	57.5 ± 11	54.5 ± 11.6	< 0.001
Female	82 (51.3)	92 (36.4)	79 (29.4)	126 (38.4)	133 (38)	57 (50.9)	< 0.001
Height (cm)	165.4 ± 8.0	166.3 ± 8.5	165.3 ± 8.2	163.5 ± 8.1	164.1 ± 8.3	162.0 ± 8.8	< 0.001
Weight (kg)	64.9 ± 12.8	68.0 ± 12.5	67.6 ± 11.5	66.3 ± 10.5	65.8 ± 11.4	63.6 ± 12.1	0.005
BMI (kg/m^2^)	23.6 ± 3.7	24.5 ± 3.5	24.7 ± 3.3	24.8 ± 3.2	24.4 ± 3.3	24.2 ± 3.5	0.001
Waist-Hip ratio	0.87 ± 0.06	0.88 ± 0.07	0.89 ± 0.07	0.91 ± 0.07	0.91 ± 0.06	0.89 ± 0.06	< 0.001
SBP (mmHg)	126.3 ± 15.6	126.1 ± 16.1	126.3 ± 15.3	126.1 ± 15.3	129.4 ± 17.3	136.1 ± 19.8	< 0.001
DBP (mmHg)	77.6 ± 10.9	77.2 ± 11.9	76.6 ± 10.1	75.5 ± 10.4	76.1 ± 11.7	78.7 ± 11.8	0.275
Pulse Pressure (mmHg)	48.7 ± 10	48.9 ± 10.3	49.7 ± 11.8	50.5 ± 12.3	53.3 ± 12.5	57.4 ± 14.2	< 0.001
Urine ACR (mg/g cr)							< 0.001
A1 (< 30)	53 (34.4)	71 (29.6)	41 (16.3)	37 (12.1)	15 (4.5)	2 (1.9)	
A2 (30 ~ 300)	47 (30.5)	76 (31.7)	86 (34.3)	110 (35.8)	98 (29.5)	20 (18.5)	
A3 (300≤)	54 (35.1)	93 (38.7)	124 (49.4)	160 (52.1)	219 (66)	86 (79.6)	
Primary kidney disease							< 0.001
GN	81 (51)	117 (46)	111 (41)	110 (34)	93 (27)	31 (28)	
DN	4 (3)	18 (7)	45 (17)	83 (25)	121 (35)	46 (41)	
HTN	2 (1)	18 (7)	67 (25)	91 (28)	95 (27)	21 (19)	
PKD	63 (39)	79 (31)	35 (13)	30 (9)	30 (9)	12 (11)	
Unclassified	10 (6)	21 (8)	11 (4)	14 (4)	11 (3)	2 (2)	
Comorbid disease							
Coronary artery disease	4 (2.5)	7 (2.8)	24 (9)	30 (9.2)	36 (10.4)	6 (5.4)	< 0.001
Peripheral vascular disease	1 (0.6)	2 (0.8)	4 (1.5)	1 (0.3)	13 (3.7)	1 (0.9)	0.010
Cerebrovascluar disease	4 (2.5)	13 (5.2)	29 (10.9)	30 (9.2)	40 (11.5)	9 (8.1)	0.004
Diabetes	19 (11.9)	46 (18.3)	73 (27.3)	118 (36.3)	156 (45)	50 (45)	< 0.001
Hypertension	126 (78.8)	217 (86.1)	241 (90.3)	305 (93.8)	331 (95.4)	105 (94.6)	< 0.001
Congestive heart failure	0 (0)	0 (0)	1 (0.4)	3 (0.9)	10 (2.9)	1 (0.9)	0.007
Arrhythmia	1 (0.6)	5 (2)	8 (3)	8 (2.5)	7 (2)	2 (1.8)	0.701
Creatinine (mg/dL)	0.71 ± 0.14	1.00 ± 0.18	1.33 ± 0.18	1.75 ± 0.27	2.68 ± 0.61	4.75 ± 1.36	< 0.001
Cystatin C (mg/L)	0.76 ± 0.15	1.00 ± 0.20	1.33 ± 0.24	1.76 ± 0.33	2.60 ± 0.52	3.89 ± 0.70	< 0.001
eGFR (mL/min/1.73 m^2^)							
MDRD	109.4 ± 20.2	73 ± 8.6	52.1 ± 4.4	37 ± 4.2	23.2 ± 4.3	11.8 ± 2.3	< 0.001
CKD-EPI cr (2009)	110.9 ± 10.4	80.7 ± 10.9	55.8 ± 5.5	38.9 ± 4.8	23.8 ± 4.7	11.9 ± 2.5	< 0.001
Hemoglobin (g/dL)	14 ± 1.4	13.9 ± 1.7	13.5 ± 1.8	12.8 ± 1.8	11.7 ± 1.5	10.6 ± 1.2	< 0.001
P (mg/dL)	3.5 ± 0.6	3.5 ± 0.6	3.5 ± 0.5	3.6 ± 0.6	3.9 ± 0.6	4.7 ± 0.7	< 0.001
hsCRP (mg/L)	1 ± 2.3	1.8 ± 3.9	2.2 ± 6.4	1.7 ± 3	3.1 ± 8.1	1.9 ± 3.8	< 0.001
Intact PTH (pg/mL)	40.2 ± 17.7	38.8 ± 14.2	53.2 ± 30.2	55.7 ± 21	92.4 ± 41.8	220 ± 118.3	< 0.001
25D (ng/mL)	15.8 ± 4.8	18.9 ± 6.7	19.3 ± 7.7	18.7 ± 7.3	18.2 ± 8.2	17.1 ± 7.8	< 0.001
1,25D (pg/mL)	30.5 ± 7.6	30.8 ± 7.3	28.5 ± 7.5	26.9 ± 5.8	23.5 ± 5.3	24.7 ± 7.4	< 0.001
Cardiac parameters							
Mean baPWV (cm/s)	1313.4 ± 189.3	1419.4 ± 239.4	1502 ± 303.6	1587 ± 329.4	1637 ± 417.6	1629.8 ± 369.2	< 0.001
LVMI (g/m^2^) (Echocardiography)	85.5 ± 23	86.2 ± 23.1	89.9 ± 23.9	96.1 ± 25.2	104.1 ± 31.2	109.6 ± 39.6	< 0.001
Coronary Ca score	44.2 ± 225.7	101.1 ± 313.1	230.7 ± 705.3	219.5 ± 599.1	252.9 ± 634.9	241.9 ± 644.6	< 0.001
Troponin T (ng/mL)	0.006 ± 0.003	0.008 ± 0.004	0.011 ± 0.006	0.016 ± 0.015	0.024 ± 0.024	0.031 ± 0.030	< 0.001
Valve calcification (Echocardiography)	12 (7.6)	31 (12.6)	39 (15.3)	75 (23.5)	75 (22.5)	30 (28)	< 0.001

^1^CKD stage classified by MDRD equation.

ANOVA or Kruskal-Wallis test for continuous variables and Chi-square or Fisher’s Exact test for categorical variables.

Numbers denote mean ± s.d. or number (%).

*Abbreviations:* GN, glomerulonephropathy; DN, diabetic nephropathy; HTN, hypertensive nephropathy; PKD, autosomal dominant polycystic kidney disease; baPWV, brachial-to-ankle pulse wave velocity; LVMI, left ventricular mass index.

## Discussion

The prevalence of CKD is increasing, as are the risk factors for CKD, such as diabetes, metabolic syndrome, and hypertension [22]. The National Health and Nutrition Examination Survey (NHANES) data from the U.S.A. show that the prevalence of CKD increased from 10% in the 1994–1998 cohort up to 13.1% in the 1999–2004 cohort [23].

CKD is a major risk factor for the development of cardiovascular complications and all-cause mortality. One large observational study reported that the risk of death increased dramatically as the GFR decreased below 60 ml/min per 1.73 m^2^[3]. The adjusted hazard ratio for death was 3.2; the eGFR ranged from 15 ml/min to 29 ml/min per 1.73 m^2^.

In a recent meta-analysis of the general population cohort, including a total of more than one million individuals [24], the risk of death was fairly constant at eGFR of 75–105 ml/min per 1.73 m^2^. However, albuminuria, which is the earliest marker of glomerular damage, was independently associated with increased mortality, even in the presence of normal GFR. This point emphasizes the importance of albuminuria as a prognostic indicator, independent of GFR stages. Therefore, unlike previous CKD cohort studies, the KNOW-CKD included subjects with albuminuria or other early markers of kidney damage and with normal or mildly decreased GFR (CKD stage 1 to 2), as well as subjects with GFR < 60 ml/min per 1.73 m^2^ (CKD stages 3 to 5). The KNOW-CKD will also analyze various outcomes of CKD based on the specific causes of CKD and based on the GFR and albuminuria categories.

Many studies have shown that racial/ethnic disparities exist in mortality and cardiovascular outcome among individuals with CKD [25-28]. Asian patients with CKD generally have a lower burden of CV disease compared with other racial groups. The KNOW-CKD, which is the first large-scale cohort study with Korean patients with CKD, aims to explore the course of renal progression and CV events and to elucidate the risk factors for renal or CV outcomes in Korean subjects with CKD.

The original MDRD Study equation [29], which was developed in 1999, was modified for creatinine assays traceable to the international reference material for creatinine [12]. However, the accuracy and the precision of the MDRD equation have been controversial issues, especially at higher GFR. The CKD-EPI equation, which uses the same four variables as the MDRD Study equation, has less bias than the MDRD Study equation [14], especially at GFR > 60 ml/min per 1.73 m^2^, and is suggested to be more accurate than the MDRD formula in Asian populations. The KNOW-CKD will employ the MDRD Study equation [12], as well as the CKD-EPI creatinine [14] and the CKD-EPI creatinine-cystatin C equations [15], to ensure the accuracy of the GFR estimation. Creatinine and cystatin C assays will be conducted in a central laboratory, using assay methods traceable to the international reference materials [16,17].

The KNOW-CKD will evaluate the complication profiles of anemia and mineral-metabolic derangements across the CKD stages. The KNOW-CKD will compare the clinical manifestations and natural course of the following four major renal etiologic subgroups: GN, DN, HTN, and PKD. Various biomarkers from the serum and urine samples will be evaluated to determine the risk prediction of adverse consequences. The genetic and epigenetic factors will be targeted. The KNOW-CKD will also evaluate the socio-economic burden and its impact on the quality of life, and the hospitalization of patients with CKD. Comparing the KNOW-CKD with other large-scale CKD cohorts through international collaboration may provide a platform for future research.

## Conclusion

As the first large-scale CKD cohort study to be established and maintained longitudinally for up to 10 years, the KNOW-CKD will help to clarify the natural course, complication profiles, and risk factors of Asian populations with CKD. Including subjects in the early stages of CKD as well as those in the advanced stages and focusing on the causes will permit insight into CKD.

## Abbreviations

GN: Glomerulonephropathy; DN: Diabetic nephropathy; HTN: Hypertensive nephropathy; PKD: Autosomal dominant polycystic kidney disease.

## Competing interests

The authors declare that they have no conflicts of interest.

## Authors’ contributions

K-H O and SKP participated in the design of the study, reviewed and collected data using e-CRF and electronic medical records system, performed the statistical analysis, and drafted the manuscript. HCP participated in the analysis and interpretation of data. HJC, DWC, KHC, SHH, THY, KL, Y-SK, WC, Y-HH, SWK, YHK, and SWK participated in the design of the study, patient enrollment, acquisition of data, analysis, and interpretation of data. BJP and JL participated in the analysis and interpretation of data. CA had substantial contributions to funding, conception and design of the study, and draft and revision of the manuscript. All authors read and approved the final manuscript.

## Pre-publication history

The pre-publication history for this paper can be accessed here:

http://www.biomedcentral.com/1471-2369/15/80/prepub
